# Complex Chromosomal Rearrangement Causes Male Azoospermia: A Case Report and Literature Review

**DOI:** 10.3389/fgene.2022.792539

**Published:** 2022-02-24

**Authors:** Yi Liang, Yingjun Xie, Shu Kong, Qianying Pan, Wenjun Qiu, Ding Wang, Mengting Li, Sisi Lin, Zihang Liu, Xiaofang Sun

**Affiliations:** ^1^ Department of Obstetrics and Gynecology, Key Laboratory for Major Obstetric Diseases of Guangdong Province, The Third Affiliated Hospital of Guangzhou Medical University, Guangzhou, China; ^2^ Key Laboratory of Reproduction and Genetics of Guangdong Higher Education Institutes, The Third Affiliated Hospital of Guangzhou Medical University, Guangzhou, China; ^3^ Department of Clinical Medicine, The Third Clinical School of Guangzhou Medical University, Guangzhou, China

**Keywords:** complex chromosomal rearrangement (CCR), azoospermia, karyotype, whole-genome sequencing, gametogenesis

## Abstract

**Background:** Male carriers of complex chromosomal rearrangements (CCRs) may have decreased fertility and usually present with azoospermia, oligospermia or teratospermia.

**Methods:** High-resolution karyotype analysis using G-banding on peripheral blood lymphocytes was performed in an azoospermic male. Copy number variations (CNVs) were detected by chromosomal microarray analysis, and genetic variations were determined by long-read nanopore sequencing with Sanger sequencing for breakpoint confirmation.

**Results:** The karyotype of the patient was 46,XY,t(4;21)(p11;p11),t(5;6;14)(p13q22;p22q22;q22), which did not involve CNVs with clinical significance. Twelve breakpoints in chromosomes 5, 6, and 14 were found by long-read nanopore sequencing. Reports on 17 males carrying CCRs with azoospermia were also reviewed.

**Conclusion:** The extent of asynaptic regions in synaptonemal complexes during pachytene and the disruption of genes involved in male gametogenesis may cause azoospermia in CCR carriers.

## Introduction

Complex chromosomal rearrangements (CCRs) are chromosomal structural abnormalities that contain at least three breakpoints on two or more chromosomes, and more than 250 CCR cases have been reported (Pellestor et al., 2011; Poot and Haaf, 2015; Aristidou et al., 2018). According to the complexity and the type of rearrangement, CCRs can generally be divided into three categories: 1) three-way rearrangements, comprising three breakpoints on three chromosomes; 2) double two-way translocations, comprising two independent simple reciprocal or Robertsonian translocations; and 3) exceptional CCRs, wherein each chromosome contains multiple breakpoints (Pellestor et al., 2011).

The majority of balanced CCR carriers have normal phenotypes but have reproductive failure, including repeated miscarriages, infertility, or multiple congenital abnormalities (MCAs) (Kim et al., 2011; Madan, 2012). Most female CCR carriers bear malformed children or have recurrent miscarriages, while male CCR carriers are diagnosed with infertility problems, often presenting with azoospermia, oligozoospermia, or teratozoospermia (Coco et al., 2004).

Approximately 50% of infertile cases can be attributed to male factors, and 10–20% of infertile men are diagnosed with azoospermia (Lee et al., 2011; Rogawski and Khafagy, 2011; Regent et al., 2020). Obstructive azoospermia (OA), caused by a blocked vas deferens with preserved spermatogenesis in the testis, occurs in 40% of men with azoospermia, whereas spermatogenetic malfunctions (nonobstructive azoospermia, NOA) occur in nearly 60% of men with azoospermia (Matzuk and Lamb, 2008; Lee et al., 2011; Ghieh et al., 2019). Genetic abnormalities, including chromosomal or gene defects, are found in approximately 30% of azoospermic men (Krausz and Cioppi, 2021). The frequency of chromosomal aberrations increases to 10–15% in azoospermic men (Matzuk and Lamb, 2008; Kuroda et al., 2020).

Molecular cytogenetic techniques such as fluorescence *in situ* hybridization (FISH) and comparative genomic hybridization (CGH) arrays, have been used to study CCRs to date, but how different CCRs cause azoospermia remains an important research question. In this study, CCR was identified in an azoospermic male using traditional cytogenetic and molecular genetic methods. The relationship between CCR and azoospermia has been discussed in combination with related literature.

## Materials and Methods

### Case Report

The patient was a 29-year-old man seeking genetic counselling with his wife due to infertility for the past 3 years. The patient was 167 cm tall and weighed 70 kg. His phenotype and intelligence were normal. No spermatozoa were found in routine semen analysis, and no microdeletions were identified in the AZF-a, AZF-b, and AZF-c genes on the Y chromosome, which are noted in the recommendations of the European Academy of Andrology (EAA) and the European Quality Monitoring Network Group (EMQN) using the Y Chromosome Deletion Detection System (Promega, Madison, WI, United States). Serum testosterone was normal at 10.94 nmol/L (normative values 4.94–33.01 nmol/L), and prolactin was 10.10 ng/ml (normative values 3.46–19.4 ng/m). Serum luteinizing hormone (LH) and follicle-stimulating hormone (FSH) were 3.01 μ/L (normative values 0.57–12.07 μ/L) and 7.24 μ/L (normative values 0.7–11.1 μ/L), respectively. The hormones and menstrual cycle of his wife were entirely normal.

The couple were not consanguineous and had no adverse family history. The patient’s family members (except his sister) underwent genetic tests after detecting his chromosomal abnormality. Written informed consent was obtained for all the tests conducted in this study.

### Chromosomal Karyotype Analysis

Peripheral blood was extracted from the patient and his family members (including his wife and his parents). Peripheral blood was cultured at 37°C for 72 h in lymphocyte culture medium (Guangzhou Dahui Biotechnology). Harvested cells were exposed to a hypotonic solution (0.075 M potassium chloride solution) and then immobilized twice with methanol/acetic acid (3:1). Chromosomes were obtained after droplet, trypsin digestion and Giemsa staining. A G-band resolution of approximately 400–550 bands was routinely analysed using MetaSystems (ZEISS, Jena, Germany) on at least 20 metaphase plates per subject. Karyotype description was based on the recommendations of the International Human Cytogenetic Nomenclature (McGowan-Jordan et al., 2020).

### Chromosomal Microarray Analysis (CMA)

Genomic DNA was extracted using the QIAamp DNA Blood Mini kit (Qiagen, Dusseldorf, Germany). DNA purity, integrity and concentration were detected by Nanodrop, agarose gel electrophoresis and Qubit, respectively. Next, the genomic DNA was hybridized with Affymetrix’s Cytoscan HD Array (Affymetrix, Santa Clara, CA, United States) following the manufacturer’s procedures. Afymetrix Chromosome Analysis Suite Software 4.2 was used for result analysis. The reported copy number variation (CNV) threshold was 50 kb, and the number of markers was 25. Public databases, such as the Database of Chromosomal Imbalance and Phenotype in Humans Using Ensembl Resources (DECIPHER; https://decipher.sanger.ac.uk/), database of genomic variants (DGV; http://dgv.tcag.ca/dgv/app/home), University of California Santa Cruz Genome Browser (UCSC; http://genome.ucsc.edu/) and Online Mendelian Inheritance in Man (OMIM; http://www.omim.org), were used to interpret the data.

### Long-Read Nanopore Sequencing and Breakpoint Verification

**FIGURE 1:**
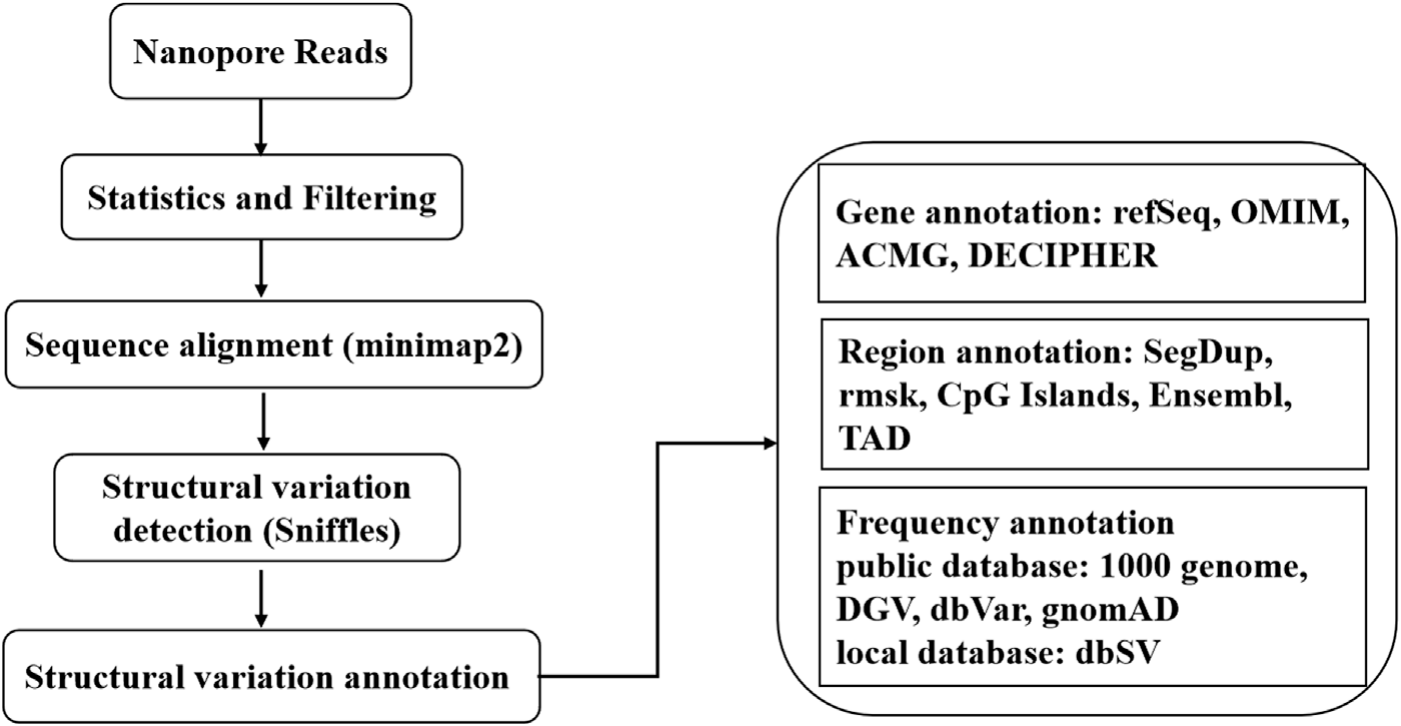
The workflow of bioinformatics analysis. The nanopore sequencer generated 3,385,816 reads containing 55.25 Gb. After filtering, 50.50 Gb with 2,964,325 reads were retained. The total number aligned to the human genome was 47.16 Gb, with 2,845,728 reads. A total of 603 SVs were annotated in ANNOVAR according to the above databases. More details are summarized in Table 1, Supplementary Table S2, S3.

A sequencing library was prepared using the SQK-LSK109 kit (Oxford Nanopore, Oxford, United Kingdom) according to the manufacturer’s procedures. The library was sequenced on PromethION (Oxford Nanopore, Oxford, United Kingdom). FASTQ files with an average quality less than 7 were filtered. Sequencing reads were aligned to the human reference genome sequence GRCh37/hg19 using minimap2. The structural variations (SVs), including deletion, insertion, duplication, inversion and translocation, were detected using Sniffles. SVs with a frequency of variation greater than 30% and a length greater than 50 bp were retained. Functional annotation of the detected and retained SVs and related genes around the breakpoint was performed in ANNOVAR according to databases such as the 1,000 Genome SV, gnomAD, DGV, dbVar, and Decipher databases. The flow chart of the bioinformatics analysis is shown in Figure 1. The translocation breakpoints were verified using Sanger sequencing on an ABI3730XL sequencer (Applied Biosystems, Foster City, CA, United States). Primer information is available in Supplementary Table S1.

### Literature Review of CCRs Associated With Azoospermia

A literature search was performed on male CCR carriers with azoospermia to provide information on the relationship and mechanisms between CCRs and azoospermia.

## Results

### Cytogenetic Investigation

**FIGURE 2:**
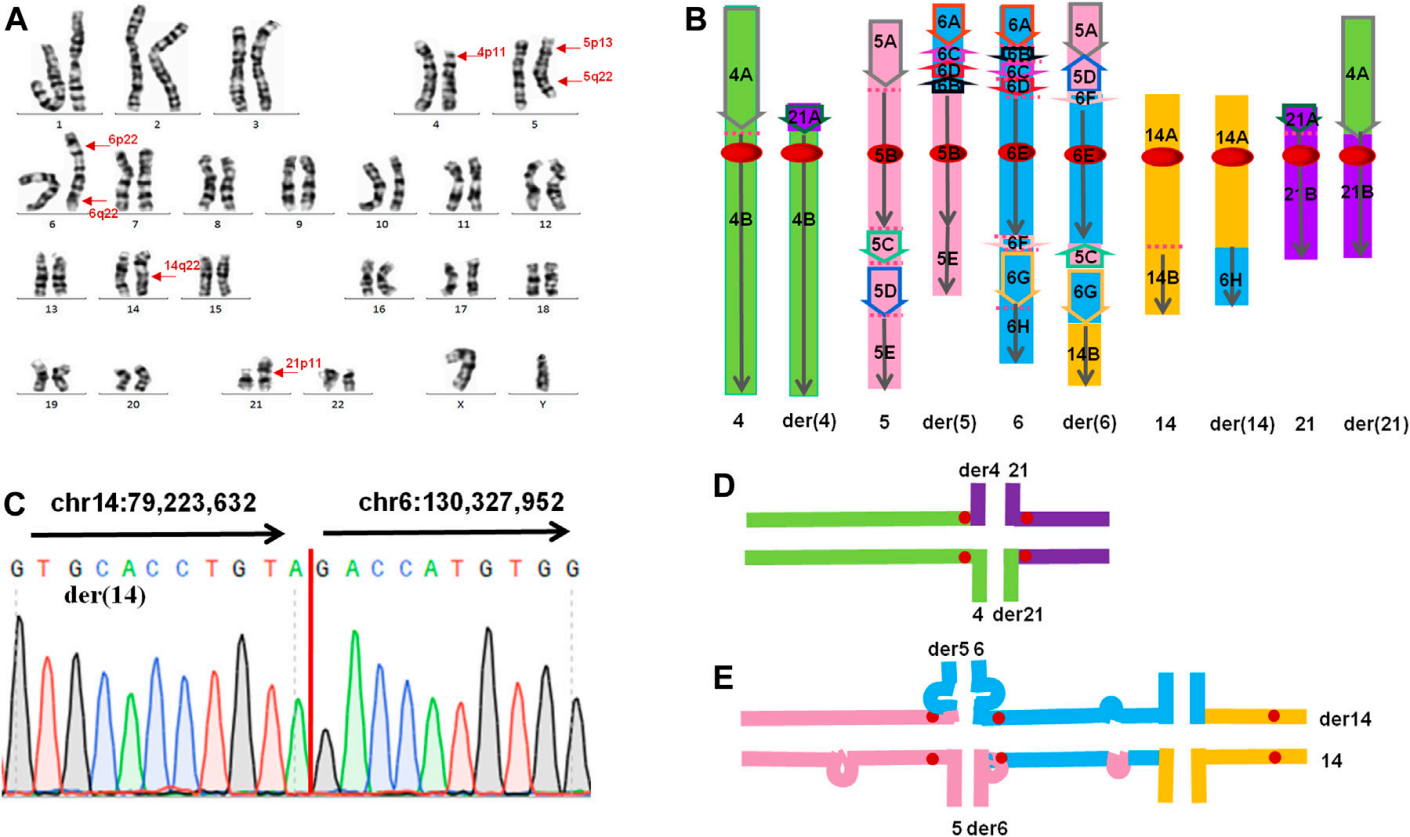
**(A)** The karyotype result of the patient. Arrows indicate the breakpoints. **(B)** The presumed pattern of variation of involved chromosomes in this CCR. The green colour represents chromosome 4, the pink colour represents chromosome 5, the blue colour represents chromosome 6, the orange colour represents chromosome 14, and the purple colour represents chromosome 21. **(C)** Sanger sequencing result on der(14). The translocation breakpoints (red line) detected by nanopore sequencing were confirmed by Sanger sequencing. Sequence directions from different chromosomes were indicated by arrows. **(D,E)** Putative chromosome pairing by complex chromosomal rearrangement during the pachytene stage of meiosis. **(D)** Schematic view of a quadrivalent configuration. **(E)** Schematic view of a hexavalent configuration. Substantial portions of the asynaptic regions in the hexavalent configuration mainly contain insertion and inversion.

G-banding chromosomal karyotype analysis revealed complex translocation involving chromosomes 4, 5, 6, 14, and 21. The karyotype of the patient was 46,XY,t(4;21)(p11;p11),t(5;6;14)(p13q22;p22q22;q22) (Figure 2A). Both parents and his wife had normal results.

### Chromosomal Microarray Analysis (CMA)

CMA of the peripheral blood for this patient was arr(X,Y)×1,(1–22)×2, and no microdeletion or microduplication with clinical significance was found. The CMA result of the patient was showed in Supplementary Figure S1.

### Long-Read Nanopore Sequencing

**FIGURE 3:**
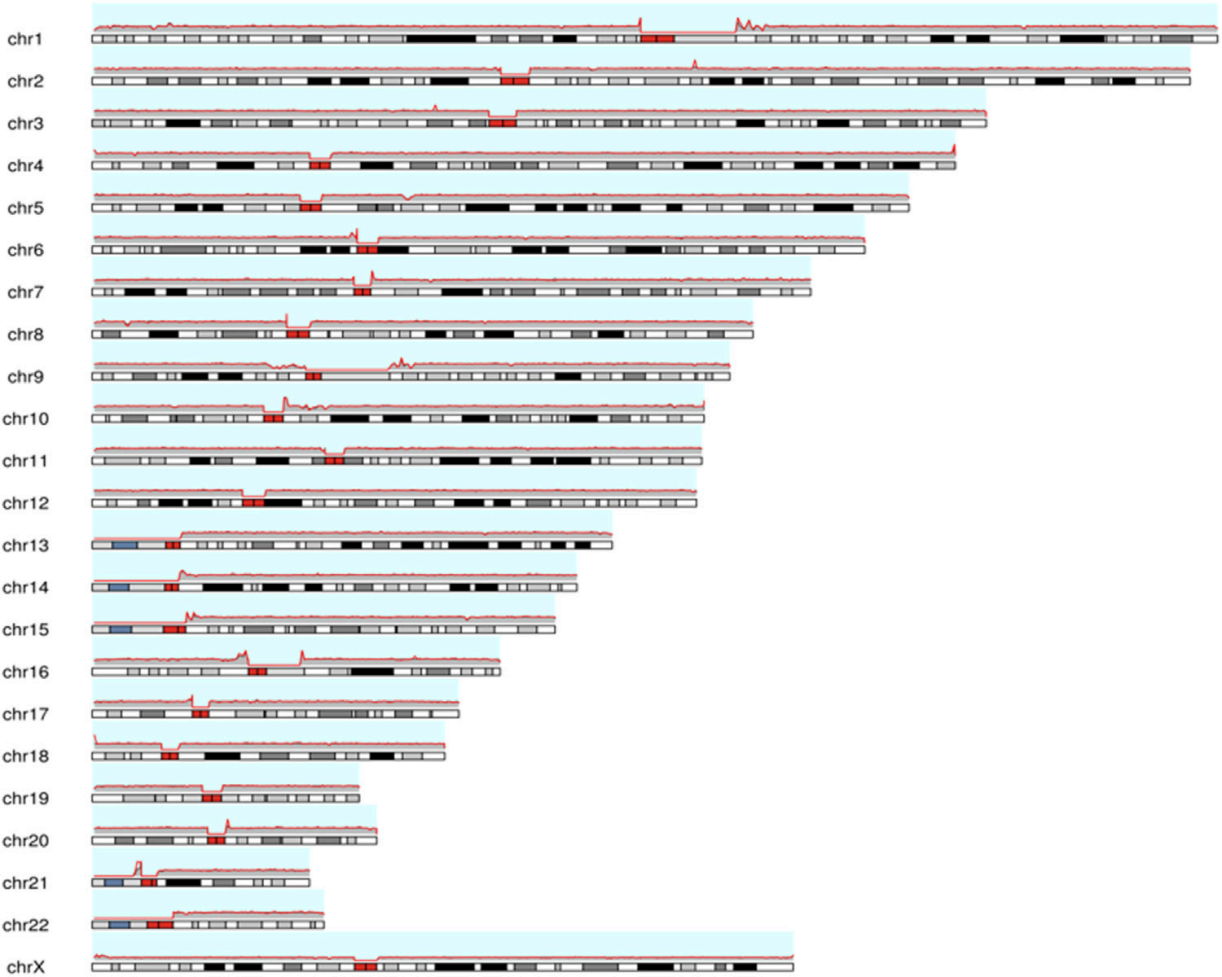
The map for chromosomal coverage through reads alignment with reference sequence using long-read nanopore sequencing. The red line represents the coverage depth of different positions in the chromosome. Cytogenetic bands are inside each chromosome, and the red bands represent the centromere of each chromosome. ChrY chromosomes were not counted in male samples. More details are showed in Supplementary Figure S2.

The total number of bases aligned to the human genome was 47.16 Gb, with a read N50 of 24.36 kb and a depth of 15.72-fold (Table 1). A total of 96.0% of the reads had no less than one alignment to the hg19 reference genome (Figure 3). The sequencing data confirmed the presence of the translocation between chromosome 5 and chromosome 6 as well as the translocation between chromosome 6 and chromosome 14. It also illustrated the presence of an insertion from segments of 5q into chromosome 6. In total, 12 breakpoints in chromosomes 5, 6, and 14 were discovered, which formed three chromosomal rearrangements. Except for the failure to detect a reciprocal translocation between chromosome 4 and chromosome 21 due to method limitations, the sequencing results of the other chromosomal breakpoints were essentially consistent with those of the karyotype results. Combining the karyotype and sequencing results, 14 breakpoints in chromosomes 4, 5, 6, 14, and 21 were detected, illustrating the complex character of this rearrangement (Figure 2). Chromosome 4, chromosome 14 and chromosome 21 contained one breakpoint, chromosome 5 had four breakpoints, and chromosome 6 had seven breakpoints (Figure 2B). Detailed information about the breakpoints and influenced genes is summarized in Table 2.

**TABLE 1:** Summary of the aligned sequences.

Sample ID	Pass reads	Mapped reads	Mapped reads Rate (%)^a^	Pass bases (bp)	Mapped bases (bp)	Mapped bases Rate (%)^b^	Depth (X)
A0024_LJJ	2,964,325	2,845,728	96.0	50,499,312,344	47,159,465,978	93.39	15.72

a Mapped Reads Rate (%): Percentage of total reads aligned to the reference and total reads qualified for quality control.

b Mapped Bases Rate (%): Percentage of total bases aligned to the reference and total bases qualified for quality control.

**TABLE 2:** Details of the breakpoints in the current study.

Chromosome	No. of breakpoints	Breakpoint position^a^	Region	Reception position	Type of SV^b^	Disrupted gene (breakpoint)	Functional region	Method
4	1	—	4p11	21p11	tra	—	—	karyotype
5	4	chr5:32,200,899	5pter-5p13.3	6p22.3	tra	*GOLPH3, MTMR12*	intergenic	sequencing
		chr5:119,701,574–123,000,823	5q23.1-5q23.2	6q16.1	ins	*PRR16, FTMT, SNX2, ZNF608, GRAMD3, PRRC1, CSNK1G3, LINC01170, FAM170A*	exonic	sequencing
		chr5:123,000,824–145,570,886	5q23.3-5q32	6p22.3	ins	*FBN2, HINT1, ACSL6, IL5, SMAD5, KLHL3, EGR1, SNHG4, IK, TAF7, SPRY4, NR3C1, PRELID2*	exonic	sequencing
6	7	chr6:22,145,014	6pter-6p22.3	5p13.3	tra	*CASC15*	ncRNA_ intronic	sequencing
		chr6:22,145,015–23,554,211	6p22.3	5p13.3	inv	*LINC00340, PRL, HDGFL1*	exonic	sequencing
		chr6:22,298,751–23,847,600	6p22.3	5p13.3	dup	*PRL, HDGFL1*	exonic	sequencing
		chr6:22,298,752–23,554,212	6p22.3	5p13.3	del	*PRL, HDGFL1*	intergenic	sequencing
		chr6:94,913,660–97,489,812	6q16.1	6p22.3	ins	*TSG1, MANEA-DT*	intergenic	sequencing
						*KLHL32*	intron	
		chr6:130,327,952	6q23.1-6qter	14q31	tra	*TMEM244, L3MBTL3*	intergenic	sequencing
14	1	chr14:79,223,632	14q31-14qter	6q23.1	tra	*NRXN3*	intron	sequencing
21	1	—	21p11	4p11	tra	—	—	karyotype

a The breakpoints were manually corrected according to the actual alignment between the sequencing results and the reference sequence.

b del, deletion; ins, insertion; dup, duplication; inv, inversion; tra, translocation.

## Discussion

According to the above results, we could infer the rearrangement as follows: four breakpoints and paracentric inversions were in the short arm of chromosome 6 as well as a reciprocal translocation between chromosomes 5p13.3 and 6p22.3. The long arm of chromosome 5 broke three times and invertedly inserted segments 5q23.1 and 5q23.2-q32 into 6q16.1 and 6p22.3, respectively. Additionally, a reciprocal translocation between 4p11 and 21p11 as well as 6q23.1 and 14q31 occurred (Figure 2B). Breakpoints 4p11 and 21p11 were located in centromeric regions with highly repetitive nucleotides and had no applicable reference sequence to be aligned on hg19. According to the UCSC database, reference sequences in these regions are formed by a series of “N”. Due to the limitations of detection in the centromere region of chromosomes, no breakpoints were identified between chromosomes 4 and 21 by long-read nanopore sequencing. According to the classification of CCRs, this case is considered a double CCR.

Within more than 250 cases related to CCRs published thus far, 161 cases concerned male CCR carriers (Olszewska et al., 2020). Approximately 75% of CCRs appear *de novo* or are inherited maternally (70% of all familial cases) (Pellestor et al., 2011; Olszewska et al., 2020). To date, most men with CCRs have serious fertility problems due to spermatogenesis disorders (Salahshourifar et al., 2009). The source of CCRs is unclear. Ionizing radiation exposure, advanced paternal age, use of immunosuppressive drugs before or during pregnancy and maternal chromosomal instability have been identified as possible risk factors for the origin of CCRs (Farra et al., 2011). The origin of CCR in the patient reported in this study is difficult to determine because the above risk factors were excluded. In addition, it has been proposed that translocations of CCRs may be formed through chromothripsis (Kloosterman et al., 2011; Kloosterman et al., 2012; Weckselblatt et al., 2015; Weckselblatt and Rudd, 2015). Most constitutional chromothripsis events occur *de novo*, and those investigations thus far have been confirmed to be of patrilineal origin. Chromothripsis is produced simultaneously by multiple DNA double-strand breaks (DSBs) that are close to each other and usually occur on multiple chromosomes simultaneously (Kloosterman et al., 2011; Kloosterman et al., 2012; Weckselblatt et al., 2015; Weckselblatt and Rudd, 2015). Alternatively, mitotic errors in early embryos (Vanneste et al., 2009) or the pulverization of micronuclei (Crasta et al., 2012) could be responsible for numerous DNA breaks. Thus, we infer that this *de novo* CCR could be due to chromothripsis.

Previous studies have suggested that CCR-related sterility is the result of spermatogenesis stagnation caused by the complex meiosis structure during meiosis (Asia et al., 2014). In the first meiosis, homologous chromosomes pair up to form synaptic complexes (SCs) and undergo meiosis recombination. (Yakut et al., 2013). These events are critical for meiosis fidelity, and defects in these processes may lead to meiosis cessation and sterility (Ferguson et al., 2008). In cross-translocated carriers, homologous chromosome pairing can theoretically be achieved by the formation of tetravalent chromosomes (trivalent chromosomes of Robertson translocation) (Kurahashi et al., 2012). However, in tetravalent chromosomes, regions around the breakpoints are often not fully synaptic (Kurahashi et al., 2012). Defects in SCs are detected by the “pachytene checkpoint”, leading to p53-independent apoptosis (Coco et al., 2004). In this case, a tetravalent configuration (Figure 2D) and a especial hexavalent configuration (Figure 2E) will be formed in pachytene. A large number of unconjugated regions (mainly in the hexavalent configuration) can activate the “pachytene checkpoint” of spermatocytes and initiate the apoptosis process. This could explain the azoospermia observed in this carrier.

In addition, the breakpoints of CCRs may interrupt the genetic structure related to male gametogenesis and interfere with spermatogenesis (Yakut et al., 2013). Gene expression is a complicated process regulated by lots of factors, including cis-acting elements and trans-acting factors at the transcription level. The cis-regulatory elements such as enhancers can act at long distances away from the transcription unit (Hu and Tee, 2017; Bylino et al., 2020). Therefore, breakpoints in the intergenic regions may disturb the interactions of the promoter and transcription unit with its cis-acting regulators by “position effects” and thus affect the expression of gene (Kleinjan and van Heyningen, 2005). All the genes affected by breakpoints in the present case are shown in Table 2. Most of these genes are protein-coding genes that are involved in intracellular transport, signal transduction, transcription regulation and tumour growth inhibition. Among them, *FTMT, ACSL6* and *FAM170A* are mainly or highly expressed in the testis. *FTMT* (OMIM 608847) is a functional ferritin targeting mitochondrial gene that plays an important role in spermatogenesis. Maccarinelli et al. (Maccarinelli et al., 2017) used knockout mice to verify that *FTMT* contributes to spermatogenesis and thus to male fertility. *ACSL6* (OMIM 604443) activates the cellular metabolism of fatty acids and is highly expressed in the testis. Hale et al. (Hale et al., 2019) demonstrated that *ACSL6* activates and integrates DHA into lecithin, which is required for normal spermatogenesis. *FAM170A* (OMIM 618401; previously *Znfd*), as a new testicular-specific gene, positively regulates the expression of heat shock genes as a nuclear transcription factor (Lei et al., 2010). Devlin et al. (Devlin et al., 2020) found that *FAM170A i*s critical to sperm head morphology, progressive sperm motility and normal spermatogenesis at the end of spermatogenesis using a knockout mouse model. Azoospermia in the present case may be also the result of disruption of these important genes.

After reviewing the literature, we found reports of 18 men with azoospermia and CCRs to date, including our patient (Table 3). Most of these studies used karyotype and FISH analyses to describe the CCRs. Our study is the only one to date that uses karyotype and long-read nanopore sequencing to characterize CCR in an azoospermic male. In 18 cases, all the chromosomes except chromosomes 17, 18, 20, and X were involved in the CCRs, and chromosome 1 was the most involved. The breakpoints 5p13, 14q31.3 and 21p11 found in our case have been reported in previous studies (Joseph and Thomas, 1982; Rodriguez et al., 1985; Lee et al., 2006; Yakut et al., 2013). Notably, breakpoint 21p11 was found in three different cases (Joseph and Thomas, 1982; Lee et al., 2006), including this one, suggesting that 21p11 may be a hotspot for CCR in azoospermic men.

**TABLE 3:** Published cases of CCRs in males with azoospermia.

No	Breakpoint	Chromosome	Method	References
1	11q22, 12q13, 21p11	11, 12, 21	karyotype	Joseph and Thomas, (1982)
2	1q42, 5p13, 10q24, 12q24	1, 5, 10, 12	karyotype	Rodriguez et al. (1985)
3	Yq11.23, 12p11.2, 12q21.2, 15q13	Y, 12, 15	karyotype + FISH	Coco et al. (2004)
4	9p22, 13q21.2, 14p13	9, 13, 14	karyotype	Sills et al. (2005)
5	9p22,13 q22, 21 p11	9, 13, 21	karyotype	Lee et al. (2006)
6	1q31, 3p14, 3p24, 3q12, 3q24, 9p21.3, 9q13, 14q13	1, 3, 9, 14	karyotype + FISH	Bartels et al. 2007
7	1q42, 4q32, 1q41, 11q23, 11q24, 4q23,11q14, 11q23	1, 4, 11	karyotype + FISH	Joly Helas et al. (2007)
8	3p26, 16q13, 8q21.2	3, 16, 8	karyotype	Salahshourifar et al. (2007)
9	3q10, 3p11.1, 14q21, 14p11	3, 14	karyotype + FISH	Kim et al. (2011)
10	9q22, 1p32, 13q32, 13q14, 4p14	9, 1, 13, 4	karyotype + FISH	Kim et al. (2011)
11	2q11.2, 19p13.2, 22p11.2	2, 19, 22	karyotype + FISH	Kim et al. (2011)
12	3p23, 3q25.3, 3p11.1, 6q27, 16q24, 12q24.3	3, 6, 16, 12	karyotype + FISH	Kim et al. (2011)
13	1p22, 3q29, 5q22	1, 3, 5	karyotype	Mahjoubi and Razazian, (2012)
14	1q43, 1q44, 10q21, 10q26.1, 14q31.3, 4q23, 4q33	1, 10, 14, 4	karyotype + FISH	Yakut et al. (2013)
15	5q14.3, 15q21, 15q26, 1p13	5, 15, 1	karyotype + FISH	Nguyen et al. (2015)
16	5q11, 7p11, 7p15, 9q12, 13p12	5, 7, 9, 13	karyotype + FISH + TUNEL	Wang et al. (2015)
17	1q42.3, 1p21, 7p14.3	1, 7	karyotype + FISH + aCGH	Olszewska et al. (2020)
18	4p11, 5p13.3, 5q23.1, 5q23.2, 5q23.3, 5q32, 6p22.3 (4 breakpoints), 6q16.1, 6q23.1, 14q31, 21p11	4, 5, 6, 14, 21	karyotype + sequencing + aCGH	the present case

In conclusion, this is the first report to date to use nanopore sequencing to characterize CCR. We systematically investigated the relationship between CCR and azoospermia using conventional cytogenetic and molecular genetic methods. The occurrence of azoospermia in male CCR carriers may be related to the degree of asynaptic regions in the SC and the destruction of genes related to gametogenesis. In addition, with the development of sequencing technology, third-generation sequencing, especially nanopore sequencing, can achieve rapid sequencing and real-time base calling of long sequences at a reasonable cost. The main limitation of this study is the sample size, which needs to be increased in further studies. In addition, the specific effects of the factors identified in this study on azoospermia need to be further studied.

## Data Availability

The datasets for this article are not publicly available due to concerns regarding participant/patient anonymity. Requests to access the datasets should be directed to the corresponding author.

## Abbreviations

CCRs, Complex Chromosomal Rearrangements; CNV, Copy Number Variation; MCAs, Multiple Congenital Abnormalities; OA, Obstructive Azoospermia; NOA, Nonobstructive Azoospermia; FISH, Fluorescence *In Situ* Hybridization; CGH, Comparative Genomic Hybridization; AZF, Azoospermia Factor; LH, Luteinizing Hormone; FSH, Follicle-Stimulating Hormone; CMA, Chromosomal Microarray Analysis; SVs, Structural Variations; DSBs, Double-Strand Breaks; DHA, Docosahexaenoic Acid.
