# Supplementary material for: Complex Chromosomal Rearrangement Causes Male Azoospermia: A Case Report and Literature Review
Source: Front Genet. 2022 Feb 24;13:792539. doi: 10.3389/fgene.2022.792539 (PMC8907855; doi:10.3389/fgene.2022.792539)
Supplement: Supplementary file 1 [file DataSheet1.docx]

**Supplementary materials**

**Table S1**. Design of PCR primers to validate breakpoints.

| Breakpoint | Sequence of Forward Primer | Sequence of Reverse Primer |
| --- | --- | --- |
| Chr5-119701574 | CAGTACCAGCCAACGGTCAG | AGGCAGTTAGCAGGCTTTGC |
| Chr5-145570886 | GCATCACCATTATGGAGCAGTC | GGTGCAGACAGGTCAAGGTC |
| Chr6-130327952 | AAGGGTGACCGTGAGGGTATC | TGTGGGCCTGTCTCAAATCC |
| Chr14-79223632 | AAGGGAAAGCGAATGCTGTT | CCTGAGGCCTAGCCTTTCCTA |

**Table S2.** Statistics of raw data and filtered data

| **Sample ID** | **Raw Bases (bp)** | **Raw Reads** | **Pass Bases (bp)** | **Pass Reads** | **Mean Length (bp)** | **N50 (bp)^a^** |
| --- | --- | --- | --- | --- | --- | --- |
| BM21A0024_LJJ | 55,247,763,665 | 3,385,816 | 50,499,312,344 | 2,964,325 | 17,035 | 24,363 |

**^a^**N50: 50% of reads were longer than this value.

**Table S3.** Statistical analysis of structural variation

| **Sample ID** |  | **DEL** | **DUP** | **INS** | **INV** | **TRA** | **Other** | **Total** |
| --- | --- | --- | --- | --- | --- | --- | --- | --- |
| BM21A0024_LJJ | Before filter | 7,384 | 1,422 | 8,696 | 276 | 104 | 165 | 18,047 |
|  | After filter | 249 | 71 | 148 | 8 | 33 | 94 | 603 |

Before filter: numbers of original structural variation; After filter: the remaining number of structural variation after filtering according to the local population structural variation frequency database.

**Figure**


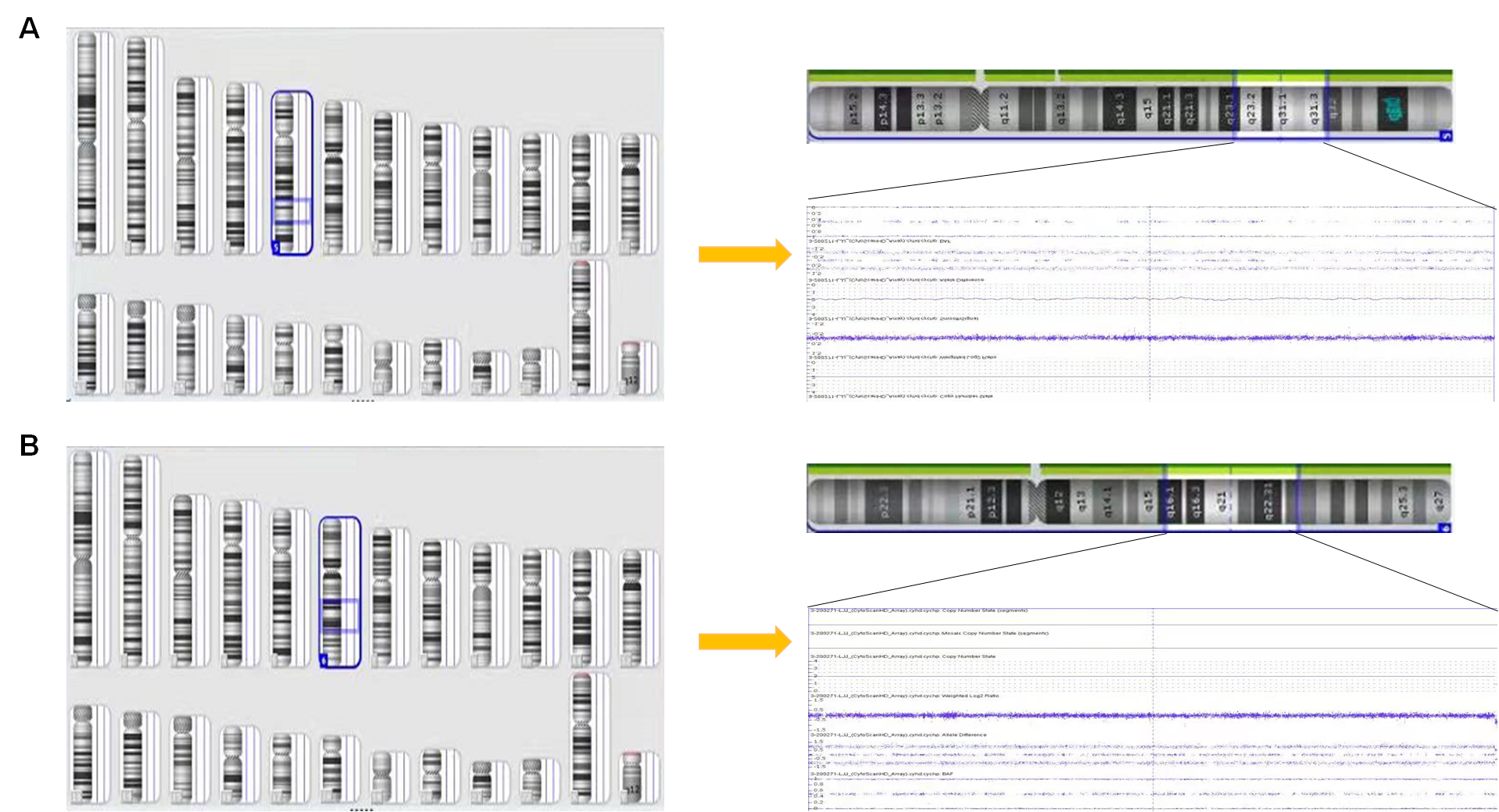


**Fig.S1.**The CMA result of the patient. (A)Detail of 5q23-q32.Three breakpoints were detected in this region using nanopore sequencing, but no microdeletion or microduplication with clinical significance were found.(B)Detail of 6q16.1-q23.Three breakpoints were detected in this region using nanopore sequencing, but no microdeletion or microduplication with clinical significance were found.


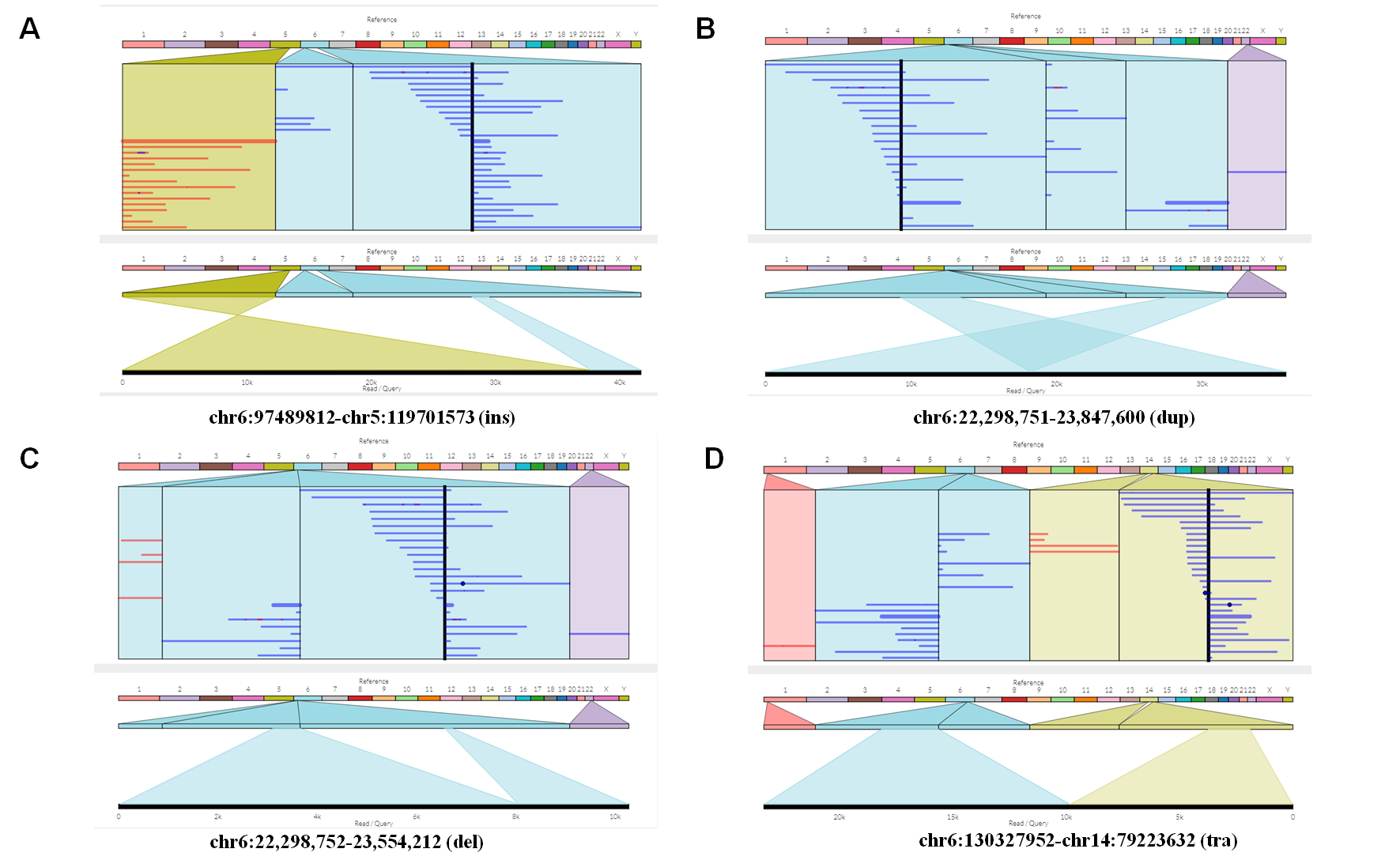


**Fig. S2.** Details of read alignment around breakpoints and SVs analysis by long-read nanopore sequencing, illustrating by Ribbon. (A) Reads alignment around chr6:97489812 and chr5:119701573. Segment 5q23.1 invertedly inserted into 6q16.1. (B) Reads alignment for chr6:22,298,751-23,847,600. A 1.5 Mb of duplication was found in 6p22.3. (C) Reads alignment for chr6:22,298,752-23,554,212. A 1.3 Mb of deletion was found in 6p22.3. (D) Reads alignment around chr6:130327952 and chr14:79223632. A reciprocal translocation between 6q23.1 and 14q31 occurred.
